# Laboratory validation and clinical performance of a saliva‐based test for monkeypox virus

**DOI:** 10.1002/jmv.28191

**Published:** 2022-10-11

**Authors:** Lao‐Tzu Allan‐Blitz, Kevin Carragher, Adam Sukhija‐Cohen, Phyllis Ritchie, Hyman Scott, Hong Li, Jeffrey D. Klausner

**Affiliations:** ^1^ Department of Medicine Brigham and Women's Hospital Boston Massachusetts USA; ^2^ Department of Population and Public Health Sciences, Keck School of Medicine University of Southern California California Los Angeles USA; ^3^ AIDS Healthcare Foundation Public Health Division Los Angeles California USA; ^4^ PS Test Palm Springs California USA; ^5^ San Francisco Department of Public Health San Francisco California USA; ^6^ Flow Health Los Angeles California USA

**Keywords:** clinical performance, diagnostics, laboratory validation, monkeypox, saliva tests

## Abstract

Improved diagnostic tests and accessibility are essential for controlling the outbreak of monkeypox. We describe a saliva‐based polymerase chain reaction (PCR) assay for monkeypox virus, in vitro test performance, and clinical implementation of that assay in Los Angeles, San Francisco, and Palm Springs, CA. Finally, using prespecified search terms, we conducted a systematic rapid review of PubMed and Web of Science online databases of studies reporting the performance of oral pharyngeal or saliva‐based tests for the monkeypox virus. The assay showed in silico inclusivity of 100% for 97 strains of monkeypox virus, with an analytic sensitivity of 250 copies/ml, and 100% agreement compared to known positive and negative specimens. Clinical testing identified 22 cases of monkeypox among 132 individuals (16.7%), of which 16 (72.7%) reported symptoms, 4 (18.2%) without a rash at the time of testing. Of an additional 18 patients with positive lesion tests, 16 (88.9%) had positive saliva tests. Our systematic review identified six studies; 100% of tests on oropharyngeal specimens from 23 patients agreed with the PCR test result of a lesion. Saliva‐based PCR tests are potential tools for case identification, and further evaluation of the performance of such tests is warranted.

## 1 INTRODUCTION

With cases of monkeypox reported from 47 countries, the World Health Organization recently declared the current spread of the infection a global emergency.^1^ Historically, the monkeypox virus has been endemic in tropical rainforest regions of Central and West Africa, with short‐lived outbreaks driven by transmission through animal‐to‐human and human‐to‐human exposures.^2^ However, the current outbreak is now spreading much more rapidly and pervasively than any previous outbreak, with a unique pattern of sexual transmission.^3^, ^4^, ^5^, ^6^ Such transmission has contributed to the disproportional burden of disease among gay, bisexual, and other men who have sex with men.^4^

The US Centers for Disease Control and Prevention has stressed the need for timely diagnosis as a primary means for outbreak control, particularly in the absence of sufficient vaccine supplies.^7^ Polymerase chain reaction (PCR) lesion testing was thought to be necessary, and the United States Food and Drug Administration has discouraged all testing apart from lesion swabs.^8^ The US Centers for Disease Control and Prevention has given similar guidance.^9^

Prior studies, however, suggest that viral DNA may be detected in saliva and oropharyngeal specimens.^2^, ^10^, ^11^ One recent report noted that 100% (*n* = 12) of patients with monkeypox had positive saliva PCR tests.^10^ Similar findings were reported among a study of seven individuals diagnosed with monkeypox in 2018 and 2019.^11^ In both studies, many individuals had positive oropharyngeal tests early in the disease course.^10^, ^11^ Thus, via early case detection, saliva testing may provide the opportunity to limit infectiousness. We, therefore, assessed the performance of a newly‐developed saliva‐based PCR assay. We further report the real‐world implementation of that assay in clinical settings.

## 2 METHODS

### 2.1 Sample preparation and PCR conditions

We describe a PCR assay of saliva for the diagnosis of monkeypox virus infection developed using DNA targets of monkeypox virus genomes (Supporting Information: 1). Patient saliva samples were collected to half of the collection kit volume (Supporting Information: 2). After collection, transport, and receipt in the laboratory, specimens were washed. For DNA extraction, 400 µl of saliva from the patient specimens were added to the Mag MAX DNA extraction kit on the KingFisher Flex Purification System (ThermoFisher Scientific Inc.). DNA amplification and detection were done using TaqMan® Real‐Time PCR (ThermoFisher Scientific Inc.). For positive specimen controls, either Acrometrix Monkeypox Thermo Control+ Human DNA Control (ThermoFisher Scientific Inc.) or Genomic DNA from a prior case of monkeypox (Monkeypox Virus; USA‐2003‐BEI‐NR‐4928) were used, depending on availability. We used two different negative specimen water controls, one during DNA extraction and an extra negative control during PCR setup to detect contamination at each step. Human RNase P was used as a human control.

The padded amplicon sequence from the target region monkeypox J1L is as follows: GTGTCTGAATCGTTCGATTAACCCAACTCATCCATTTTCAGATGAATAGAGTTATCGATTCAGACACATGCTTTGAGTTTTGTTGAATCGATGAGTGAAGTATCATCGGTTGCACCTTCAGATGC.

The PCR process began with 2 min of Uracil‐DNA glycosylation hold at 25°C, allowing mis‐primed or nonspecific targets to degrade, followed by 15 min of reverse transcription at 50°C, and 2 min of activation at 95°C. Subsequently, 40 cycles of denaturation and annealing/extension occurred over 3 and 1 s intervals at 95°C and 60°C, respectively. For our internal control, we used detection of the RNaseP sequence after PCR to ensure adequate extraction and amplification in each sample. To prevent mismatches in PCR result, the samples were plated in a checkerboard pattern in triplicate—meaning three wells were dedicated to one sample. All positive samples with cycle threshold values ≥34 were repeated for confirmation. The data were processed using either QuantStudio Flex Software version 1.5.1 or Design and Analysis Software version 2.4.3 (ThermoFisher Scientific Inc.).

We report the microbiological inclusivity of all strains with genome sequences available from two different clades: Clade I (Central Africa Clade) and Clade II (West African Clade). We further report the analytic specificity of the assay for monkeypox virus compared to other members of the orthopoxvirus genus as well as other nonorthopoxvirus genera. Subsequently, using 20 replicates of a positive control within pooled negative saliva (oral saliva matrix) specimens, we report the limit of detection, defined as the lowest concentration providing a positive result for 100% of replicates. Using two known positive specimens as well as 20 known negative specimens, we report the in vitro agreement between those specimens and our assay. For measures of agreement, at least two different operators and instruments were used on three separate days at three different concentrations to assess reproducibility. Assay validation and clinical use was conducted in accordance with the United States Clinical Laboratory Improvement Act guidelines.^12^

We reviewed deidentified patient records among individuals presenting for monkeypox virus testing at saliva collection sites in California, and, where available, concordance between saliva and lesion PCR tests (Monkeypox (Orthopox) DNA, PCR Test; Labcorp). Cycle threshold values for lesion PCR results were only available for tests performed in Los Angeles. Advarra institutional review committee exempted the analysis of deidentified data from institutional review (Pro00065270).

### 2.2 Literature review

Finally, we conducted a systematic rapid review of the literature on PubMed and Web of Science databases to assess the performance of saliva tests in comparison to PCR of lesion swabs. We used the following predefined search terms: “monkeypox” AND (“diagnosis” OR “diagnostic”) AND (“saliva” OR “sputum” OR “throat” OR “pharyn*”). We further evaluated the references of all articles identified and searched preprint servers for forthcoming publications. We included articles that reported the results of any oropharyngeal or saliva PCR tests for monkeypox in humans. We excluded review articles, studies among primates, and studies not in English. We then conducted a narrative review of the studies, reporting individual study‐level summary data given the degree of heterogeneity within studies precluded a formal meta‐analysis.

## 3 RESULTS

The PCR saliva assay had an in silico inclusivity of 100% for all (*n* = 97) strains from the two different clades. The assay was specific to the orthopoxvirus genus, but not to the monkeypox virus, as the assay also detected cowpox and rabbitpox, but did not detect nonorthopoxvirus genera. The analytic sensitivity was 250 copies/ml, the lowest dilution for which all (*n* = 20) replicates were positive (Table 1). Further, there was 100% agreement compared to known positive and negative specimens over three separate days, performed by two different operators.

**Table 1:** In vitro limit of detection for saliva‐based PCR for human monkeypox via serial dilutions

Copies/ml	Replicates	Cycle threshold value	Interpretation	% Viral positivity
0	1	Undetermined	Negative	0.0%
	2	Undetermined	Negative	
	3	Undetermined	Negative	
31.25	1	Undetermined	Negative	33.3%
	2	35.7	Positive	
	3	Undetermined	Negative	
62.5	1	34.1	Positive	66.7%
	2	34.11	Positive	
	3	Undetermined	Negative	
125.0	1	36.34	Positive	66.7%
	2	Undetermined	Negative	
	3	34.8	Positive	
**250.0**	**1**	**34.8**	**Positive**	**100%**
	**2**	**34.6**	**Positive**	
	**3**	**35.7**	**Positive**	
500.0	1	34.7	Positive	100%
	2	32.6	Positive	
	3	34.1	Positive	
1000.0	1	33.0	Positive	100%
	2	32.6	Positive	
	3	33.4	Positive	
2000.0	1	31.9	Positive	100%
	2	31.9	Positive	
	3	31.5	Positive	
Controls				
Positive Control 1	1	17.8	Positive	100%
Positive Control 2	1	28.7	Positive	100%
Positive Control 3	1	17.5	Positive	100%
Negative Control 1^a^	1	Undetermined	Negative	0.0%
Negative Control 2^a^	1	Undetermined	Negative	0.0%
Negative Control 3^a^	1	Undetermined	Negative	0.0%
Negative Extra Control 1^b^	1	Undetermined	Negative	0.0%
Negative Extra Control 2^b^	1	39.4	Negative	0.0%
Negative Extra Control 3^b^	1	Undetermined	Negative	0.0%

*Note*: Bold values indicate the selected threshold for the limit of detection.

Abbreviation: PCR, polymerase chain reaction.

^a^ Negative control during DNA extraction.

^b^ Negative control during PCR set up.

Clinical testing from three testing sites in Los Angeles County, CA, identified 22 cases of monkeypox among 132 individuals screened (16.7%). Of those 22 patients, 16 (72.7%) reported symptoms, and 4 (18.2%) did not have a rash at the time of testing, while one patient reported being asymptomatic (Table 2). We did not have data on the reported symptom status for five patients. Table 3 shows the results of saliva and lesion tests among 30 patients. In all, 16 (88.9%) of 18 patients with positive lesion tests had a positive saliva test, and 11 (100%) of 11 patients with a negative lesion test had a negative saliva test. One patient with a negative saliva test had an inconclusive lesion test.

**Table 2:** Characteristics of 22 cases of monkeypox identified via saliva‐based testing at three publicly accessible testing sites in Los Angeles

Case ID	Test result	Cycle threshold value	Gender	Symptoms reported	Known exposure	No. days between exposure and symptom onset	No. days between symptom onset and positive saliva test
1	Positive	27	Male	Lesions, headache, fever, sore throat	Yes	16	1
2	Positive	24	Male	No symptoms	Yes	‐	‐
3	Positive	18	Male	Lesions, painful lesions and/or blisters, headache, fever, muscle pain, sore throat, hot and cold flashes	Yes	5	8
4	Positive	22	Male	Lesions, headache, fever, muscle pain, sore throat, hot and cold flashes	No known exposure	‐	8
5	Positive	32	Male	Lesions, headache, muscle pain, sore throat, hot and cold flashes	Suspected exposure	‐	15
6	Positive	15	Male	Sore throat, hot and cold flashes	Suspected exposure	‐	4
7	Positive	23	Male	Lesions, painful lesions and/or blisters, headache, fever, muscle pain, hot and cold flashes	No known exposure	‐	3
8	Positive	16	Male	Lesions, headache, fever, sore throat, hot and cold flashes	Yes	8	2
9	Positive	28	Male	External lesions (hands, face, arms, legs, genital), internal lesions (oral, rectal), headache, fever	Yes	3	9
10	Positive	21	Male	External lesions (hands, face, arms, legs, genital), headache, fever, chills and hot flashes, diarrhea	No known exposure	‐	5
11	Positive	31	Male	Fever, muscle pain	No known exposure	‐	8
12	Positive	25	Male	Lesions	Yes	‐	10
13	Positive	31	Male	‐	‐	‐	‐
14	Positive	23	Male	‐	‐	‐	‐
15	Positive	28	Male	‐	‐	‐	‐
16	Positive	17	Male	Lesions, painful lesions and/or blisters, headache, fever, muscle pain, sore throat, hot and cold flashes	Yes	3	35
17	Positive	17	Male	Headache, fever, muscle pain, sore throat, hot and cold flashes	Suspected exposure	7	13
18	Positive	22	Male	Lesions, fever, muscle pain	Suspected exposure	5	4
19	Positive	21	Male	‐	‐	‐	‐
20	Positive	16	Male	‐	‐	‐	‐
21	Positive	20	Male	Headache, muscle pain, sore throat	Yes	6	8
22	Positive	18	Male	Lesions	‐	‐	‐

**Table 3:** Concordance results of saliva and lesion PCR tests among patients testing for monkeypox virus infection in Los Angeles and San Francisco, CA

Patient ID	City	Age (years)	Gender	Ethnicity	Symptoms reported	Saliva PCR	Saliva PCR cycle threshold value	Lesion PCR	Lesion PCR cycle threshold value	Days between saliva test and lesion test
LA1	Los Angeles	29	Male	White/Hispanic/Latino	Penile discharge, penile bumps, urethral irritation	Positive	28.9	Positive	25.6	6
LA2	Los Angeles	28	Male	White/Hispanic/Latino	White spots on penis	Positive	30.0	Positive	29.1	3
LA3	Los Angeles	38	Male	White/Hispanic/Latino	Blister Lesions on right leg and pelvis	Positive	28.4	Positive	28.6	2
LA4	Los Angeles	34	Male	Hispanic/Latino	Blisters in mouth and on wrists	Positive	28.0	Positive	34.2	5
LA5	Los Angeles	50	Male	Hispanic/Latino	Penile discharge, genital rash	Negative		Negative		4
LA6	Los Angeles	20	Male	Black	Penile and right forearm lesions, swollen lymph nodes in genital region, muscle aches	Positive	26.3	Positive	27.0	3
LA7	Los Angeles	31	Male	Hispanic/Latino	Pruritic painless rash on penis	Negative		Negative		4
LA8	Los Angeles	43	Male	White	Muscle and joint pain, fever, rectal pain, pimple‐like blisters on body	Positive	22.6	Positive	26.6	3
LA9	Los Angeles	38	Male	White/Hispanic/Latino	Rash and open sores on anus with swelling and bleeding	Positive	26.7	Positive	17.9	0
LA10	Los Angeles	37	Male	Black	Painful pruritic rash on hands/genitals	Positive	20.3	Positive	18.0	0
LA11	Los Angeles	32	Male	Black/Hispanic/Latino	Pruritic painful rash on anogenital region, “flu”‐like symptoms	Positive	26.6	Positive	21.6	2
LA12	Los Angeles	28	Male	White/Hispanic/Latino	Exposed to monkeypox, painless nonpruritic rash on back	Negative		Inconclusive		0
LA13	Los Angeles	42	Male	Hispanic	Penile lesions	Positive	34.3	Positive	33.5	3
SF1	San Francisco	43	Male	Hispanic/Latino	Cutaneous lesions	Positive	31.4	Positive		0
SF2	San Francisco	50	Male	White	Cutaneous lesions	Negative		Negative		0
SF3	San Francisco	27	Male	White	Cutaneous lesions	Negative		Negative		0
SF4	San Francisco	51	Male	White	Cutaneous lesions	Positive	19.2	Positive		0
SF5	San Francisco	29	Male	Hispanic/Latino	Cutaneous lesions	Negative		Negative		0
SF6	San Francisco	40	Male	Black	Cutaneous lesions	Negative		Negative		0
SF7	San Francisco	26	Male	White	Cutaneous lesions	Positive	32.6	Positive		0
SF8	San Francisco	48	Male	Asian	Cutaneous lesions	Negative		Positive		0
SF9	San Francisco	29	Male	Asian	Cutaneous lesions	Negative		Positive		0
SF10	San Francisco	50	Male	Hispanic/Latino	Cutaneous lesions	Negative		Negative		0
SF11	San Francisco	25	Male	White/Black/Hispanic	Cutaneous lesions	Negative		Negative		0
SF12	San Francisco	40	Male	Hispanic/Latino	Cutaneous lesions	Positive	20.3	Positive		0
SF13	San Francisco	55	Male	White	Cutaneous lesions	Positive	29.8	Positive		0
PS1	Palm Springs	56	Male	White	Painful lesions on genitals	Positive	20.5	Positive		1
PS2	Palm Springs	47	Male	White	Lesion in mouth	Negative		Negative		2
PS3	Palm Springs	50	Male	White	Headache and lesion on back	Negative		Negative		1
PS4	Palm Springs	25	Male	Hispanic	Painful lesion on arm	Negative		Negative		2

Abbreviation: PCR, polymerase chain reaction.

Our systematic rapid review identified 16 reports, of which 6 met our inclusion criteria. Thornhill et al., however, did not report numeric values for the total number of oral swabs PCR tests performed, and thus were excluded. One further report was found from the references of the identified articles. Among those 6 studies (all case series), there were 292 total patients, 24 of whom had tests performed on oropharyngeal or saliva specimens (Table 4). In all included studies, the results of oral fluid specimen tests were positive among 100% of patients with concomitant positive lesion swabs for monkeypox virus.

**Table 4:** Results of a systematic rapid review reporting positivity among saliva‐based tests for human monkeypox

References	Year	Country	No. cases	No. oropharyngeal specimens tested	Type of oropharyngeal specimen	Gene detected	No positive saliva tests	% Positive
Mileto et al.^21^	2022	Italy	1	1	Oropharyngeal swab	1. RealStar Orthopoxvirus PCR Kit 1.0 (Altona Diagnostics GmbH) 2. Monkeypox‐specific Generic Real‐Time PCR Assay within TNF Alpha Gene	1	100%
Patel et al.^22^	2022	UK	197	1	Tonsillar abscess	Not reported	1	100%
Piero‐Mestres et al.^10^	2022	Spain	12	12	Saliva	1. Commercial Orthopox generic real‐time PCR assay (Lightmix Roche Diagnostics) 2. Monkeypox‐specific Generic Real‐Time PCR Assay within TNF Alpha Gene	12	100%
CDC^9^	2003	USA	71	2	Oropharyngeal and nasopharyngeal swabs	Not reported	2	100%
Antinori et al.^5^	2022	Italy	4	1	Saliva	1. RealStar Orthopoxvirus PCR Kit (Altona Diagnostics GmbH) 2. Monkeypox‐specific Generic Real‐Time PCR Assay within TNF Alpha Gene^13^	1	100%
Adler et al.^11^	2022	UK	7	7	Upper respiratory tract swabs	1. Generic pan‐orthopox virus PCR^14^ 2. Monkeypox‐specific assay detecting VETF and Rp018 genes^15^	7	100%
Total			292	24			24	100%

## 4 DISCUSSION

We report the performance and use of a saliva‐based PCR test for the monkeypox virus. We supplemented our report of the assay performance with a systematic rapid review of the literature to summarize the performance of saliva‐based tests compared to lesion swabs.

Laboratory analysis demonstrated strong agreement between the saliva‐based test and known positive and negative specimens. Based on genetic sequence analysis from the published sequences (Supporting Information: 1), all strains should be detected by the assay. Genomic analysis from clinical samples will be important to confirm those results in vivo. When implemented into clinical practice, 22 cases of monkeypox were diagnosed. Notably, among those with positive saliva tests, 16 had a clinical disease, 1 was asymptomatic, and 4 did not have a rash or lesions at the time of testing. Those findings are of particular importance given the potential utility of saliva‐based tests to help detect monkeypox earlier in the time course of the illness than lesion‐based tests. A further 30 patients had lesion swabs collected concurrently, among which we demonstrated high concordance with saliva tests.

One report from Belgium identified asymptomatic cases via rectal testing.^16^ Additionally, a preprint report from the Democratic Republic of the Congo reported detection of monkeypox virus DNA from a throat swab of individuals with prodromal symptoms.^17^ Thus, oral fluid and/or saliva‐based tests may have the potential for earlier detection of cases. Earlier case identification would likely result in behavior change to reduce infectiousness and earlier treatment to decrease lesion development. Further work should directly compare the performance of saliva‐based tests to lesion swabs during different stages of infection.

One additional consideration beyond earlier detection is the concern that if the virus can be detected before lesion development, it may also be transmittable before lesion development. Previous work has similarly suggested that viral shedding at various anatomic sites may contribute to transmission. Delayed viral detection may reflect protracted infectiousness.^11^ In addition, from prior outbreaks of monkeypox human‐to‐human transmission through respiratory droplets has been suggested among a small subset of cases.^2^

Two further benefits of saliva‐based testing are worth considering. The current outbreak has consistently presented with anogenital lesions.^11^, ^18^, ^19^ Rapid and accessible testing of anogenital lesions may be more challenging than saliva‐based tests, given that patients will require privacy to collect anogenital specimens, in contrast to walk‐up or drive‐through saliva testing centers for SARS‐CoV‐2. Further, adapting SARS‐CoV‐2 testing sites for monkeypox virus saliva testing will rapidly expand testing accessibility and capacity across the country. Beyond convenience, however, the predominance of anogenital lesions in conjunction with data from contact tracing efforts have strongly suggested sexual transmission,^4^, ^5^ particularly among gay, bisexual, and other men who have sex with men.^4^ Urgent work is needed to control the outbreak, and saliva‐based tests may be a crucial component of those efforts.

One challenge in understanding the performance of saliva‐based tests is the heterogeneity with which such tests have been used in published case series.^5^ Our systematic rapid review, however, demonstrated 100% agreement with lesion PCR testing.^5^, ^10^, ^11^, ^20^, ^21^, ^22^ That performance may not reflect the true performance of saliva‐based tests given the small overall sample size and that all included studies were case series. From one large study not included in our review as there was no denominator for the number of saliva‐based tests from which we could calculate the percent agreement with lesion testing, false negative tests were reported compared to PCR testing of seminal fluid.^4^ Thus, an accurate and precise estimate of saliva‐based test performance in comparison to PCR of lesion swabs is urgently needed.

Our study has several limitations. Regarding the clinical sensitivity of the assay, robust measures could not be assessed given the overall small sample size of those with a comparator test. The clinical accuracy of saliva‐based tests for the monkeypox virus will be determined by comparisons of the saliva‐based test results to larger samples of other reference standards of infection (i.e., positive viral lesion tests or serological conversion). With regard to the systematic rapid review, the quality of the studies included was poor and heterogeneous, thus again a definitive determination of the performance of saliva‐based tests was not possible. Therefore, our findings should be viewed as a call to action in the development and comparison of saliva‐based specimen testing.

## 5 CONCLUSION

We report the laboratory and clinical performance of a saliva‐based PCR test for the monkeypox virus. Supplementing that report, we systematically reviewed the literature for all reports of saliva‐based tests for the monkeypox virus. Our findings provide evidence that saliva‐based tests may be a viable testing method for the monkeypox virus and may identify cases earlier than lesion‐based tests, warranting further evaluation of saliva‐based assays.

## AUTHOR CONTRIBUTIONS

All authors contributed substantially to this study. Lao‐Tzu Allan‐Blitz developed the search criteria for the systematic review, analyzed the data, wrote the first draft of the manuscript, and contributed to revisions. Kevin Carragher assisted in data collection and project oversite as well as data analysis and revisions of the manuscript. Adam Sukhija‐Cohen assisted in project oversight and implementation as well as revision of the manuscript. Phyllis Ritchie assisted in project oversight, data collection, data analysis, and revisions of the manuscript. Hyman Scott assisted in project oversight, data collection, data analysis, and revisions of the manuscript. Hong Li oversaw laboratory procedures, assisted with data collection and data analysis, and contributed to revisions of the manuscript. Jeffrey D. Klausner oversaw all project operations, assisted in data collection and data analysis, and contributed to revisions of the manuscript.

## CONFLICT OF INTEREST

H. L. is the laboratory director for Flow Health. The remaining authors declare no conflict of interest.

## Supporting information

Supplementary information.

Supplementary information.

## Data Availability

Data are available upon request.
