# Supplementary figures and images for: Laboratory validation and clinical performance of a saliva‐based test for monkeypox virus
Source: J Med Virol. 2022 Oct 11;95(1):e28191. doi: 10.1002/jmv.28191 (PMC10091791; doi:10.1002/jmv.28191)

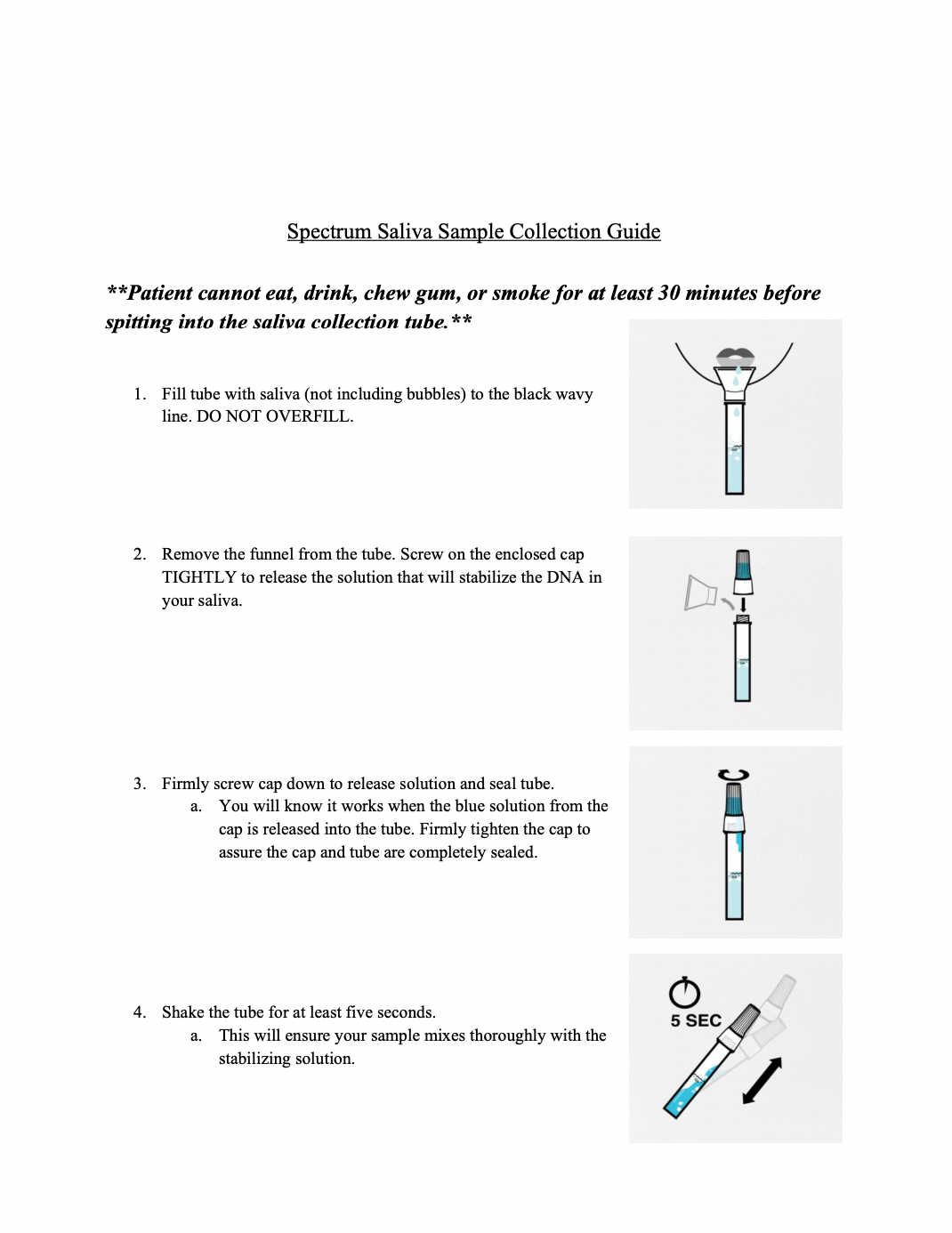

Supplement: Supplementary file 2 — Supplementary information. [file JMV-95-0-s002.png]
